# Substance use disorder in young adults with stroke: clinical characteristics and outcome

**DOI:** 10.1007/s13760-023-02317-8

**Published:** 2023-07-15

**Authors:** Hoda Ibrahim Rizk, Rehab Magdy, Khadiga Emam, Mona Soliman Mohammed, Alshaimaa M. Aboulfotooh

**Affiliations:** 1 Department of Public Health and Community Medicine, Kasr Al-Ainy Faculty of Medicine, Cairo University, Cairo, Egypt; 2 Department of Neurology, Kasr Al-Ainy Faculty of Medicine, Cairo University, Cairo, Egypt

**Keywords:** Stroke, Substance use disorder, Tramadol, Cannabis, Heroin

## Abstract

**Objective:**

Stroke incidence among young adults has risen in the last decade. This research attempts to determine the effect of substance use disorder (SUD) on the clinical characteristics of stroke, mortality, outcome after IV thrombolysis, and functional dependency after 1 month among young adults.

**Methods:**

Through a retrospective study, data were extracted from the electronic medical records of stroke in young adults admitted to intensive care units in Kasr Al-Ainy Hospital (February 2018–January 2021). The National Institute of Health Stroke Scale (NIHSS) and the Modified Rankin Scale were documented at the onset and after 1 month.

**Results:**

The study included 225 young adults with stroke (median age of 40, IQR: 34–44). Only 93 young adults (41%) met the criteria of SUD. Anabolic steroid use disorder was significantly associated with cerebral venous thrombosis (*P*-value = 0.02), while heroin use disorder was significantly associated with a hemorrhagic stroke (*P*-value = 0.01). Patients with tramadol, cannabis, and cocaine use disorders had significantly more frequent strokes in the posterior than the anterior circulation. Patients with heroin use disorders had significantly higher mortality than those without heroin use disorders (*P*-value = 0.01). The risk of poor outcomes was doubled by alcohol or heroin use disorder, while it was tripled by cocaine use disorder (*P*-value = 0.01 for each).

**Conclusion:**

Forty-one percent of young adults diagnosed with a stroke had SUD, with a relatively higher posterior circulation involvement. Increased mortality was associated with heroin use disorder more than other substances. Poor stroke outcome was associated with alcohol, heroin, and cocaine use disorders.

## Introduction

The worldwide prevalence of stroke in 2019 was 101.5 million people, whereas that of ischemic stroke was 77.2 million, that of intracerebral hemorrhage was 20.7 million, and that of subarachnoid hemorrhage was 8.4 million [1].

Strokes may affect people at the peak of their productive lives. Despite its enormous impact on countries' social and economic development, this growing crisis has not received much attention [2]. Although diagnosis and stroke management methods have significantly improved, undetermined etiology remains the main cause in this age group [3].

Substance use disorder (SUD), a risk factor for stroke, is a growing threat worldwide among young adults. Prevention is the primary treatment strategy. Disseminating awareness and promoting research on young stroke adults is essential to mitigate the burden of stroke [4].

As epidemiological studies of stroke in Egypt are scarce, accurate knowledge of stroke among young adults regarding the risk factors, especially SUD, its prevalence, and studying its effects on stroke outcome is crucial to establishing public health strategic and preventive plans to mitigate the stroke burden [5].

The primary aim of this study was to describe clinical and radiological characteristics in an Egyptian cohort of young stroke adults in relation to SUD. A secondary aim was to study the impact of SUD on mortality, stroke outcome following IV thrombolysis, and functional dependency outcome at 1 month of follow-up.

## Methods

This retrospective observational study included all young adults with stroke consecutively admitted to the stroke unit of Cairo University Hospital between February 2018 and January 2021. Data from all young adults with stroke (age < 45 years) admitted to our institution were prospectively recorded. We exclude incomplete records and stroke-related head trauma cases. The ethical committee of the Neurology department-Faculty of Medicine-Cairo University approved the study.

These data were extracted from electronic stroke registries: demographics and medical conditions, such as hypertension, diabetes mellitus, dyslipidemia, ischemic heart disease, arrhythmias, obesity (body mass index ≥ 30), and others. Diagnosis of SUD was made according to the Diagnostic and Statistical Manual of Mental Disorders-5th edition (DSM-5) [6] based on either urine drug testing or medical record history.

The severity of the neurological deficit on admission was evaluated by the National Institutes of Health stroke scale (NIHSS) [7] and The Modified Rankin Scale (mRS) [8].

At the initial presentation, all patients were investigated using brain computed tomography (CT) and magnetic resonance imaging (MRI). Further imaging modalities were performed case-by-case based on the physician's decision as computed tomography angiography (CTA) or magnetic resonance venography (MRV). Lesions in middle cerebral or anterior cerebral vascular territories denote anterior circulation involvement. In contrast, lesions in the territory of the vertebral artery, basilar artery, posterior cerebral artery, or their penetrating arteries were defined as posterior circulation [9].

Intravenous r-TPA was administered according to the American Heart Association/American Stroke Association (AHA/ASA) guidelines for the early management of acute ischemic stroke [10]. Brain CT was re-performed 48 h after the r-TPA bolus administration for patients with ischemic stroke or any neurological deterioration.

### Study outcomes

- Outcome after IV thrombolysis: was determined as favorable or unfavorable at 48 h after rtPA bolus administration. The favorable outcome was defined as a complete resolution of the neurological deficit or an improvement in the mRS score from baseline by two or more points 24 h after stroke onset.
- Survival or mortality.
- Functional stroke outcome wa***s*** evaluated as either good or poor at a 1-month follow-up. A good outcome at a 1-month following stroke is defined as mRS scores of 0–2, whereas scores of 3–6 represent a poor outcome [11].

### Statistical analysis

Data entries were carried out using SPSS (Statistical Package for Social Science) version 23.0 (IBM, SPSS, USA). The normality distribution of the data was tested by using the Shapiro–Wilk test. Categorical variables were expressed in numbers and percentages. Not normally distributed continuous variables were expressed as median and interquartile ranges. Chi-square and Fisher’s exact tests were used to compare the characteristics of stroke between young patients with SUD versus stroke in young adults without SUD; Mann–Whitney was used as appropriate for comparing and measuring the disability resulting from the stroke in young adults with SUD versus stroke in young adults without SUD. *P*-value < 0.05 was considered significant.

## Results

**Fig. 1:**
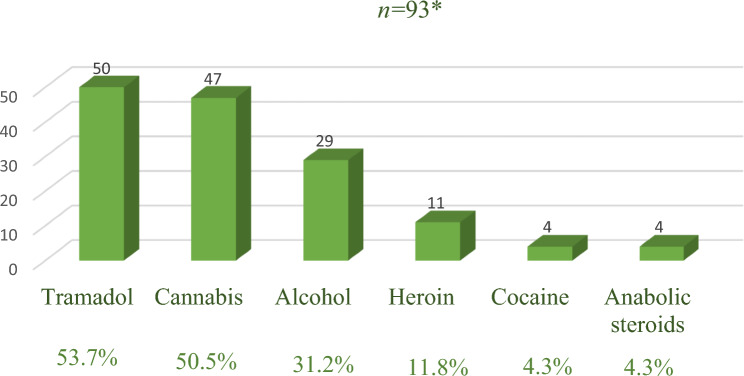
Frequencies of different substances abused by young stroke adults. *42 young adults were abusing more than one substance

Two-hundred-and-twenty-five strokes were identified in young adults (139 males & 86 females), with a median age of 40 (IQR: 34–44). Two hundred-six patients had an arterial stroke (179 with ischemic stroke, 13 with ICH, and 14 with SAH), while venous stroke was documented in 19 patients. Among the study population, 93 young adults (41%) met the criteria of SUD. The most frequently abused substance among the abusers was tramadol (54%), followed by cannabis (51%) (Fig. 1).

The frequency of some vascular risk factors, including diabetes, hypertension, dyslipidemia, and obesity, was significantly lower in adults with SUD than in those without SUD. Detailed demographics and vascular risk factors are illustrated in Table 1.

**Table 1:** Demographic and clinical characteristics of the study population

	Substance use disorder (*n* = 225)		*P*-value
	Yes (*n* = 93)	No (*n* = 132)	
Age [Median (IQR)]	39 (33–42)	41 (37–44)	0.01
Gender			
Male (*n* = 139)	70 (50.3%)	69 (49.4%)	0.01
Female (*n* = 86)	23 (26.7%)	63 (73.3%)	
Diabetes			
Yes (*n* = 36)	6 (16.7%)	30 (83.3%)	0.01
No (*n* = 189)	87 (46%)	102 (54%)	
Hypertension			
Yes (*n* = 52)	9 (17%)	43 (83%)	0.01
No (*n* = 173)	84 (48.6%)	89 (51.4%)	
Dyslipidemia			
Yes (*n* = 41)	9 (22%)	32 (78%)	0.01
No (*n* = 184)	84 (45.7%)	100 (54.3%)	
Cardiac arrhythmia			
Yes (*n* = 18)	4 (22%)	14 (78%)	0.09
No (*n* = 207)	89 (43%)	118 (57%)	
IHD			
Yes (*n* = 15)	3 (20%)	12 (80%)	0.08
No (*n* = 210)	90 (43%)	120 (57%)	
Peripheral vascular disease			
Yes (*n* = 7)	1 (14.3%)	6 (85.7%)	0.24
No (*n* = 218)	92 (42%)	126 (58%)	
HIV			
Yes (*n* = 2)	2 (100%)	0 (0%)	0.17
No (*n* = 223)	91 (41%)	132 (59%)	
Obesity			
Yes (*n* = 28)	0 (0%)	28 (100%)	0.01
No (*n* = 197)	93 (47%)	104 (53%)	

*IHD* ischemic heart disease, *HIV* human immunodeficiency virus

### Substance use disorder and stroke pathology

It was found that cannabis use disorder was significantly associated with the arterial type of stroke, while anabolic steroid use disorder was significantly associated with the venous type (Table 2). Also, 60% of adults with heroin use disorder had a hemorrhagic stroke versus 40% who developed an ischemic type (*P* 0.01). Other substances showed no statistically significant difference between stroke types (Table 3).

**Table 2:** Relation between different types of substances and type of stroke

	Stroke type (*n* = 225)		*P*	RR	95% CI
	Arterial (*n* = 206)	Venous (*n* = 19)			
Alcohol					
Yes (*n* = 29)	29 (100%)	0 (0%)	0.08	1.1	1.06–1.16
No (*n* = 196)	177 (90%)	19 (10%)			
Tramadol					
Yes (*n* = 50)	48 (96%)	2 (4%)	0.2	2.6	0.6–11.6
No (*n* = 175)	158 (90%)	17 (10%)			
Cannabis					
Yes (*n* = 47)	47 (100%)	0 (0%)	0.02	1.11	1.1–1.2
No (*n* = 178)	159 (91%)	19 (9%)			
Heroin					
Yes (*n* = 11)	10 (90%)	1 (10%)	0.9	0.9	0.11–7.58
No (*n* = 214)	196 (92%)	18 (8%)			
Cocaine					
Yes (*n* = 4)	4 (100%)	0 (0%)	1	1.1	1–1.13
No (*n* = 221)	202 (91%)	19 (9%)			
Anabolic steroids					
Yes (*n* = 4)	1 (25%)	3 (75%)	0.02	0.026	0.003–0.26
No (*n* = 221)	205 (92%)	16 (8%)

**Table 3:** Comparison of ischemic versus hemorrhagic stroke types among young adults in relation to different substances

	Arterial stroke (*n* = 206)		*P*-value	RR	95% CI
	Ischemic (*n* = 179)	Hemorrhagic (*n* = 27)			
Alcohol					
Yes (*n* = 29)	25 (86%)	4 (14%)	0.9	0.9	0.3–3
No (*n* = 177)	154 (87%)	23 (13%)			
Tramadol					
Yes (*n* = 48)	39 (81%)	9 (19%)	0.2	0.6	0.2–1.3)
No (*n* = 158)	140 (89%)	18 (11%)			
Cannabis					
Yes (*n* = 47)	41 (87%)	6 (13%)	1	1	0.4–2.7
No (*n* = 159)	138 (87%)	21 (13%)			
Heroin					
Yes (*n* = 10)	4 (40%)	6 (60%)	0.01	0.1	0.02–0.3
No (*n* = 196)	175 (89%)	21 (11%)			
Cocaine					
Yes (*n* = 4)	3 (75%)	1 (25%)	0.4	0.4	0.04–4.4
No (*n* = 202)	176 (87%)	26 (13%)			
Anabolic steroids					
Yes (*n* = 1)	1 (100%)	0 (0%)	1	1.2	1.1–1.2
No (*n* = 205)	178 (87%)	27 (13%)			

### Substance use disorder and stroke location

Patients with tramadol, cannabis, and cocaine use disorders had significantly more frequent strokes in the posterior than the anterior circulation (Table 4).

**Table 4:** Relation between different types of substances and location of stroke

	Ischemic and ICH (*n* = 192)		*P*-value	RR	95%CI
	Anterior (*n* = 124)	Posterior (*n* = 68)			
Alcohol					
Yes (*n* = 27)	13 (48%)	14 (52%)	0.1	0.5	0.2–1.1
No (*n* = 165)	111 (67%)	54 (33%)			
Tramadol					
Yes (*n* = 43)	19 (44%)	24 (56%)	0.01	0.3	0.2–0.7
No (*n* = 149)	105 (70%)	44 (30%)			
Cannabis					
Yes (*n* = 43)	16 (37%)	27 (63%)	0.01	0.2	0.1–0.5
No (*n* = 149)	108 (73%)	41 (27%)			
Heroin					
Yes (*n* = 8)	6 (75%)	2 (25%)	0.6	1.7	0.3–8.5
No (*n* = 184)	118 (64%)	66 (36%)			
Cocaine					
Yes (*n* = 3)	0 (0%)	3 (100%)	0.04	2.9	2.4–3.5
No (*n* = 189)	124 (66%)	65 (34%)			
Anabolic steroids					
Yes (*n* = 1)	1 (100%)	0 (0%)	1	1.5	1.4–1.8
No (*n* = 191)	123 (64%)	68 (36%)			

Regarding SAH, 10 cases were identified as aneurysmal SAH (6 with posterior communicating artery aneurysms, 3 with middle cerebral artery aneurysms, and one with anterior cerebral artery aneurysms), and 4 cases had peri-mesencephalic SAH. Mycotic aneurysms were identified in only two cases with heroin use disorder.

Among patients with SUD, intracranial stenosis was documented by CTA in nine cases (3 cases with cocaine use disorder, other 3 with cannabis cocaine use disorder, 2 cases with heroin use disorder, and only one had alcohol use disorder). All these cases had intracranial stenosis involving the anterior circulation, except for two cases (one with heroin use disorder and another with alcohol use disorder), in which posterior circulation was involved.

### Substance use disorder and stroke disability

Patients with alcohol, heroin, and cocaine use disorders had significantly higher NIHSS and mRS scores at the onset. On the other hand, patients with tramadol and cannabis use disorders were significantly associated with higher mRS scores but not with NIHSS (Table 5).

**Table 5:** Relation between different types of substances and stroke disability at onset

	Stroke at onset (*n* = 225)			
	NIHSS	*P*	Modified Rankin scale	*P*
Alcohol				
Yes (*n* = 29)	12 (9–16)	0.01	4 (3–5)	0.01
No (*n* = 196)	8 (6–13)		3 (3–3)	
Tramadol				
Yes (*n* = 50)	9 (7–16)	0.06	3 (3–3)	0.01
No (*n* = 175)	9 (6–14)		3 (3–3)	
Cannabis				
Yes (*n* = 47)	9 (7–14)	0.82	3 (3–4)	0.02
No (n = 178)	9 (5–14)		3 (3–4)	
Heroin				
Yes (*n* = 11)	18 (16–21)	0.01	5 (4–5)	0.01
No (*n* = 214)	9 (6–13)		3 (3–3)	
Cocaine				
Yes (*n* = 4)	20 (18–21)	0.01	5 (5–5)	0.01
No (*n* = 221)	9 (6–14)		3 (3–4)	
Anabolic steroids				
Yes (*n* = 4)	8 (6–12)	0.75	3 (3–4)	0.7
No (*n* = 221)	9 (6–14)		3 (3–4)	

### Substance use disorder and IV thrombolysis outcome

Seventy-four young patients were eligible for r-TPA administration (19 were with SUD, and 55 were without SUD). Out of 19 patients with SUD, only one did not improve after receiving r-TPA. This patient met the criteria of SUD for three substances (alcohol, tramadol, and cannabis). There was no statistically significant difference between illicit substances regarding IV thrombolysis outcome.

### Substance use disorder and mortality outcome

Among the whole study population, only eight cases died. Patients with heroin use disorders had significantly higher mortality than those without heroin use disorders (P-value = 0.01) (Table 6).

**Table 6:** Mortality outcome of stroke among young adults with different types of substances

	Stroke outcome *n* = 225		*P*-value	RR	CI
	Survival (*n* = 217)	Mortality (*n* = 8)			
Alcohol					
Yes *n* = 29	29 (100%)	0 (0%)	0.2	1.04	1.01–1.07
No *n* = 196	188 (95.9%)	8 (4.1%)			
Tramadol					
Yes *n* = 50	47 (94%)	3 (6%)	0.2	0.4	0.1–1.9
No *n* = 175	170 (97.1%)	5 (2.9%)			
Cannabis					
Yes *n* = 47	45 (95.7%)	2 (4.3%)	0.7	0.7	0.2–4
No *n* = 178	172 (96.4%)	6 (3.4%)			
Heroin					
Yes n = 11	9 (81.8%)	2 (18.2%)	0.01	0.13	0.02–0.7
No *n* = 214	208 (97.2%)	6 (2.8%)			
Cocaine					
Yes *n* = 4	4 (100%)	0 (0%)	1	1.03	1.01–1.06
No *n* = 221	213 (96.4%)	8 (3.6%)			
Anabolic steroids					
Yes *n* = 4	4 (100%)	0 (0%)	1	1.03	1.01–1.06
No *n* = 221	213 (96.4%)	8 (3.6%)			

### Substance use disorder and functional outcome at a 1-month follow-up

Poor outcome was significantly higher among patients with alcohol, cocaine, and heroin use disorder. The risk of poor outcomes among patients with alcohol or heroin use disorder was double that among the non-alcoholic or non-heroin use disorders (*P*-value = 0.01 for each). All patients with cocaine use disorder (4 patients) had a poor outcome, being at three times at risk of having a poor outcome than non-cocaine use disorder (*P*-value = 0.01) (Table 7).

**Table 7:** Functional stroke outcome at 1 month of follow-up among young adults with different types of substances

	Stroke outcome *n* = 225		*P*-value	RR	95% CI
	Good	Poor			
Alcohol					
Yes *n* = 29	12 (41%)	17 (59%)	0.01*	2	1–3
No *n* = 196	130 (66%)	66 (34%(			
Tramadol					
Yes *n* = 50	30 (60%)	20 (40%)	0.8	1	0.7–1.6
No *n* = 175	112 (64%)	63 (36%)			
Cannabis					
Yes *n* = 47	29 (62%)	18 (38%)	0.9	1	0.7–1.6
No *n* = 178	113 (64%)	65 (36%)			
Heroin					
Yes *n* = 11	2 (18%)	9 (82%)	0.01*	2	1.6–3.6
No *n* = 214	140 (66%)	74 (34%)			
Cocaine					
Yes *n* = 4	0 (0%)	4 (100%)	0.01*	3	2–4
No *n* = 221	142 (65%)	79 (35%)			
Anabolic steroids					
Yes *n* = 4	3 (75%)	1 (25%)	1	1	0.1–4
No *n* = 221	139 (63%)	82 (37%)			

*P-value is significant

## Discussion

With the lack of detailed information about the effect of SUD among young adults on stroke characteristics and outcomes in Egypt, this study provided a better understanding of SUD-induced stroke in Egyptian young adults.

The co-existence of different comorbidities and SUD was investigated in this study; these comorbidities represent the main modifiable risk factors for stroke. It was noticed that most young stroke adults with different comorbidities developed stroke without SUD. This might denote that SUD, even without other comorbidities, is sufficient to be a risk factor for stroke.

Similar to our results, Chang, Münster [12] showed that anabolic androgenic steroids increased the risk of cerebral venous thrombosis. It is well-established that anabolic steroids cause an increase in prothrombin fragments and thrombin/antithrombin complexes, which leads to cerebral venous thrombosis [13, 14].

The present study showed that all cannabis abusers developed an arterial stroke predominantly of ischemic type (86%) involving the posterior circulation. This is in line with Middlekauff, Cooper [15], who found that cannabis-induced multifocal intracranial stenosis mostly involved the posterior circulation.

In contrast, the hemorrhagic type predominated over the ischemic stroke in heroin abusers. This could be explained by pyogenic arteritis or the rupture of mycotic aneurysms induced by heroin [16].

In light of the previous studies' agreement that cocaine-related stroke can be of any type and may happen in every brain region [16], the current study showed a significant preference for posterior circulation involvement among patients with cocaine use disorder. Potent cerebral vasospasm, cerebral vasculitis, and bradykinin-mediated endothelial dysfunction all have been demonstrated as consequences of cocaine abuse [17].

In 2016, a meta-analysis revealed that heavy alcohol consumption increases the risk for the hemorrhagic type, either ICH or SAH [18]. Regarding the ischemic events, alcohol-induced atrial fibrillation mainly contributes to cerebrovascular events [19], as the effects of alcohol on hemostatic and fibrinolytic factors are still doubtful [20]. However, we found no association between alcohol use disorder and any specific stroke type.

For tramadol, one of the most commonly abused substances by Egyptian youths [21, 22], data about its pathophysiological mechanisms of causing stroke is not settled yet [23]. Contrary to classic opioids, tramadol inhibits serotonin and norepinephrine reuptake, provoking autonomic hyperactivity like tachycardia, hypertension, and cardiac arrhythmia, which all may contribute to cerebrovascular events [24].

Only 74 patients in this study had received r-TPA during stroke management. Twenty percent of abusers received r-TPA, whereas 40% of the non-abusers were eligible for r-TPA, which poses a question of whether substance abuse might affect the issues related to the eligibility for the usage of r-TPA in the treatment protocol. However, no statistically significant differences in stroke outcome after IV thrombolysis between different illicit substances.

Mortality among young stroke patients was estimated to be 4.5% at 30-day follow-up by Rutten-Jacobs, Arntz [25]. Yet, the mortality rate reported by the current study (3.5%) was relatively lower than the previously mentioned study. In general, high mortality outcomes among SUD patients might be attributed to the adverse cardiac effects of illicit substances [26]. Mahtta, Ramsey [27] reported an increased risk of premature atherosclerotic cardiovascular diseases induced by all substances included in the current study. However, in the present study, heroin was the only substance associated with significantly higher mortality, which might be attributed to the predominantly hemorrhagic stroke type associated with heroin use disorder. It is well-known that intracerebral hemorrhage has a higher case fatality rate than ischemic stroke [28, 29].

The present study observed poor functional outcomes among patients with alcohol, heroin, and cocaine use disorders, using the Modified Rankin Scale after 1 month of stroke onset. It is well-established that baseline NIHSS strongly predicts functional outcomes [30–32]. As previously reported in this study, alcohol, heroin, and cocaine were significantly associated with higher NIHSS scores at the onset; these substances would be expected to be significantly associated with a poor outcome. These findings disagree with Sheikh Andalibi, Rezaei Ardani [33], who did not find a significant difference in functional dependency between patients with SUD versus non-SUD.

This present study had some limitations that should be mentioned. Some stroke patients refrained from seeking emergency treatment at hospitals for fear of infection during the COVID-19 pandemic; this influenced the number of young stroke cases collected in this study. Secondly, the study followed the stroke outcome only for 1 month, missing the opportunity to assess the outcome (recurrence, disability, and mortality) for extended periods post-stroke, hoping that future research will overcome these limitations.

## Conclusion

This study concluded that 41% of young adults diagnosed with a stroke had SUD. Posterior circulation stroke was higher among patients with tramadol, cannabis, and cocaine use disorder. Cerebral venous thrombosis showed a higher frequency among anabolic steroid use disorders, whereas the frequency of hemorrhagic strokes was higher among heroin use disorder.

High mortality was associated with heroin use disorder more than other substances. Poor stroke outcome was associated with alcohol, heroin, and cocaine use disorders.

The current study emphasizes the importance of screening for SUD, especially among young adults with stroke, to improve the outcome.

## Data Availability

Authors report that the datasets used and/or analyzed during the current study are available from the corresponding author upon reasonable request.
